# Single-cell RNA sequence presents atlas analysis for chondrocytes in the talus and reveals the potential mechanism in coping with mechanical stress

**DOI:** 10.3389/fcell.2022.1047119

**Published:** 2022-11-10

**Authors:** Tianrui Wang, Junjie Wang, Zewen Sun, Lu Zhang, Chenghao Yu, Haibo Zhao, Mingyue Yan, Shenjie Sun, Zhenhao Ye, Yingze Zhang, Tengbo Yu

**Affiliations:** ^1^ Department of Orthopaedic Surgery, The Affiliated Hospital of Qingdao University, Qingdao, China; ^2^ Qingdao Medical College, Qingdao University, Qingdao, China; ^3^ Medical Research Center, Institute of Orthopaedics and Traumatology, The Affiliated Hospital of Qingdao University, Qingdao, China; ^4^ LC-Bio Technologies, Co., Ltd., Hangzhou, China

**Keywords:** single-cell RNA sequencing, talus, cartilage, mechanical stress, chondrocyte

## Abstract

Chondrocytes are indispensable for the function of cartilage because they provide the extracellular matrix. Therefore, gaining insight into the chondrocytes may be helpful in understanding cartilage function and pinpointing potential therapeutical targets for diseases. The talus is a part of the ankle joint, which serves as the major large joint that bears body weight. Compared with the distal tibial and fibula, the talus bears much more mechanical loading, which is a risk factor for osteoarthritis (OA). However, in most individuals, OA seems to be absent in the ankle, and the cartilage of the talus seems to function normally. This study applied single-cell RNA sequencing to demonstrate atlas for chondrocyte subsets in healthy talus cartilage obtained from five volunteers, and chondrocyte subsets were annotated. Gene ontology and Kyoto Encyclopedia of Genes and Genomes pathway enrichment analyses for each cell type, cell–cell interactions, and single-cell regulatory network inference and clustering for each cell type were conducted, and hub genes for each cell type were identified. Immunohistochemical staining was used to confirm the presence and distribution of each cell type. Two new chondrocyte subsets were annotated as MirCs and SpCs. The identified and speculated novel microenvironment may pose different directions in chondrocyte composition, development, and metabolism in the talus.

## Introduction

Articular cartilages refer to the connective tissues that cover the distal bone surfaces, such as the femur, tibia, talus, and humerus. These connective tissues, one kind of hyaline cartilage, are mesenchyme-sourced (Bhosale and Richardson, 2008). The major functions of cartilage include the lubricating motion between the bone ends and bearing and absorbing internal and external mechanical loading, owing to its biomechanical property (Carballo et al., 2017). Articular cartilage tissue is composed of two major elements, chondrocytes and the extracellular matrix (ECM), and the latter consists of water, aggrecan, and collagen type 2. The ECM provides tensile strength and compressive resilience and accounts for 98 to 99 percent of the volume of cartilage, leaving the remaining space for the sparsely distributed chondrocytes (Ge et al., 2012; Goldring and Goldring, 2016). However, the chondrocytes are indispensable for the function of cartilage because of the ECM they provide (Carballo et al., 2017). Therefore, gaining insight into the chondrocytes is of significance for understanding the cartilage function and pinpointing potential therapeutical targets for diseases.

To date, several studies have reported chondrocyte subsets such as proliferative chondrocytes (ProCs), prehypertrophic chondrocytes (preHTCs), hypertrophic chondrocytes (HTCs), cartilage progenitor cells (CPCs), effector chondrocytes (ECs), regulatory chondrocytes (RegCs), and homeostatic chondrocytes (HomCs) (Jeon et al., 2017; Ji et al., 2019; Prein et al., 2016; Saito et al., 2010; St-Jacques et al., 1999). However, fewer studies have focused on the chondrocyte microenvironment in the talus. The talus is a part of the ankle joint, which serves as the major large joint that bears body weight (Kleipool and Blankevoort, 2010). Moreover, compared with the distal tibial and fibula, the talus bears much more mechanical loading. It is shown that several factors and pathways associated with inflammation are activated upon mechanical stress, such as, TNF-α, Wnt, microRNA, and oxidative stress pathways, which participate in the promotion of inflammatory progression and the stimulation of enzymes for ECM degradation, including metalloproteinases and aggrecanases. These cytokines, pathways, and enzymes have been verified to impair the normal microenvironment in cartilage and contribute to the osteoarthritis (OA) process (Wong and Carter, 2003; Zhu et al., 2020). However, in most individuals, OA seems to be absent in the ankle, and the cartilage of the talus seems to function normally. Therefore, it is an ideal candidate to study the chondrocyte constitution in normal cartilage.

The sequencing technique has experienced remarkable development in the first two decades of the 21st century. Compared with next-generation sequencing, single-cell RNA sequencing (scRNA-seq) allows researchers to explore the tissue more profoundly, including identifying cell subsets, cell–cell interactions, and development relationships, as well as differential expressed genes (DEGs) and genetic transcription conditions. In this study, cartilage tissues of the talus were obtained from healthy individuals, and scRNA-seq was conducted. Afterward, chondrocyte subsets, their functions, development relationship, and cell–cell interactions were identified or speculated, and genetic transcription conditions were also determined. This study aims to gain deep insight into normal cartilage tissue and chondrocytes in the talus.

## Materials and methods

### Volunteers and samples

**FIGURE 1:**
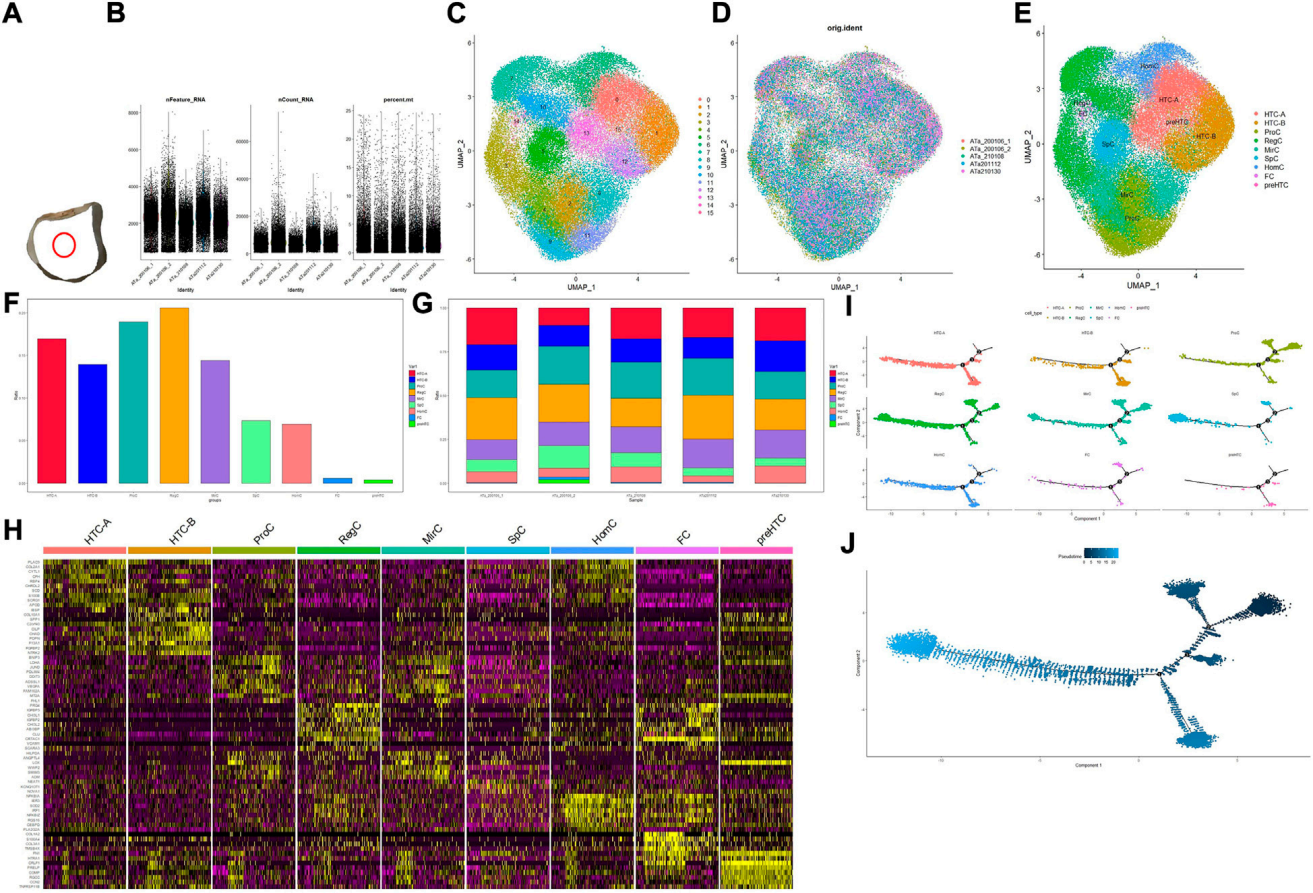
Overall process and results for scRNA-seq. **(A)** Location of the acquired cartilage sample; **(B)** basic information on quality control; **(C)** result for clustering; **(D,G)** distribution of the cartilage sample in the cluster and cell type, respectively; **(E)** result for cell-type annotation; **(F)** percentage for each cell type; **(H)** heatmap for top 10 markers in each cell type, and the color of the point represents the expression levels; **(I)** pseudotime analysis for different cell types; **(J)** development analysis for the overall developmental line, and the color of the point represents the trajectory order.

Five cartilage tissues from the trochlear surface of the talus that are in contact with the distal tibia were obtained from five volunteers who had suffered from fatal accidents. None of the five volunteers had been diagnosed with rheumatoid, gout, or ankle trauma, as well as any diseases that might affect the alignment of the hip-knee-ankle. All cartilage tissues were from the surface that was in contact with the distal end of the tibia, and the location of cartilage samples is shown in Figure 1A. Demographic information for the five volunteers is shown in Table 1. This study was approved by the Institutional Review Board and Ethics Committee of the Affiliated Hospital of Qingdao University, and all donors or their family members signed written informed consent.

**TABLE 1:** Demographic characteristics for the volunteers enrolled.

Sample number	Sex	Age (years)	Height (cm)	Weight (kg)
ATa_200,106_1	Male	65	172	58
ATa_200,106_2	Male	69	155	65
ATa_210,108	Male	56	160	60
ATa201112	Male	57	160	55
ATa210130	Male	58	170	65

### Cartilage resection and preparation for single-cell suspensions

After dislocating the talus from the joint capsule, fresh cartilage tissues were resected, and the depth of incision was controlled to include each layer of the cartilage and avoid the subchondral bone. Afterward, samples were put into a sterile culture dish that contained phosphate-buffered saline (PBS), and then, they were cut into pieces (0.5 mm^3^) and irrigated with PBS twice. All of these processes were performed on ice to maintain the 4°C temperature. Subsequently, these pieces were put into a dissociation solution (0.2% collagenase Ⅱ and 0.25% Trypsin-EDTA) that was kept in a 37°C water bath and shaken at 100 rpm for 20 min simultaneously in order to acquire single cells. Digestion was suspended using PBS with 10% fetal bovine serum. The resulting cell suspension was filtered by using a 70–30-μm stacked cell strainer and centrifuged at 300 g for 5 min at 4°C. The Miltenyi ^®^ Dead Cell Removal Kit (MACS 130-090-101) was used in order to remove dead cells. Lastly, it was centrifuged twice at 300 g for 3 min at 4°C for resuspension. The overall cell viability should be more than 85% and was determined by trypan blue exclusion. Single-cell suspensions were counted *via* the Countess II Automated Cell Counter with a level of 700–1,200 cells/μl.

### Chromium 10X genomics library and sequencing

The single-cell suspension obtained previously was then loaded onto 10X Chromium to capture single cells based on the manufacturer’s instructions of the 10X Genomics Chromium Single-Cell 3′ kit (V3). A standard protocol was followed in amplifying cDNA and constructing the library. The library was sequenced on an Illumina NovaSeq 6000 sequencing system (paired-end multiplexing run, 150 bp) by LC-Bio Technology Co., Ltd. (Hangzhou, China) at a depth of at least 20,000 reads per cell.

### Cell clustering

Illumina bcl2fastq software (version 2.20) was applied to transform the sequencing results into the FASTQ format. The Cell Ranger pipeline (https://support.10xgenomics.com/single-cellgene expression/software/pipelines/latest/what-is-cell-ranger, version 4.0.3) was used to generate the three files (barcode, matrix, and feature) for each sample. scRNA-seq data were converted to gene symbol using the Ensembl genome GRCh38/GRCm38 reference genome. Afterward, the Seurat (version 3.1.1) R package was used to create a Seurat object and experience quality control, reducing dimension and clustering. The quality control process included removing genes expressed in fewer than three cells, removing cells that expressed fewer than 500 genes, and reducing the percentage of mitochondrial DNA-derived genes to less than 25%. The function CellCycleScoring was used to evaluate the impact of the cell cycle phase on clustering and annotation. The UMAP method was used in visualizing the cluster. Marker genes were screened if they were expressed in more than 10% of the cells in each cluster, and the average log2 Fold Change (FC) was greater than 0.25 using the function FindMarkers.

### Immunohistochemical staining

The IHC process of talus cartilage included the following steps: fixation in 4% buffered paraformaldehyde for no less than 2 d, decalcification with EDTA for about 2–3 months until needles could be stuck into the tissue without obvious obstruction, paraffin embedment and sectioning, dewaxation and rehydration of paraffin sections with xylene and gradient alcohol, blockage of endogenous peroxidase activity with 3% H_2_O_2_ for 10 min, serum sealing with 3% BSA for 30 min at normal temperature, antigen retrieval with pepsin digestion, primary antibody incubation overnight at 4°C and secondary antibody incubation for 50 min at room temperature, DAB color reaction, and counterstaining the nucleus with the hematoxylin stain solution for about 3 min. An upright microscope (NIKON Eclipse ci) and imaging system (NIKON digital sight DS-FI2) were used to form and enlarge images using white light.

### Gene ontology and Kyoto Encyclopedia of Genes and Genomes pathway enrichment analyses

GO and KEGG are two major resources that are used to explore the potential function and pathway for multiple genes. Metascape (http://metascape.org/gp/#/main/step1), an online analyzing tool for enrichment analysis, was used to conduct GO and KEGG pathway enrichment analyses for DEGs in each cluster. *p* < 0.05 was regarded as statistically significant. GO terms were presented as figures based on the number of top 20 markers in each cluster or *p*-value, while KEGG terms were listed based on the *p*-value only.

### Cell-type annotation

DEGs with a relatively high log2fc value that could be discriminated on a scattered diagram were chosen as marker genes for each cluster. By comparing the marker genes with those provided by Fuchou Tang et al. (Ji et al., 2019), the cell type was annotated. GO and KEGG pathway enrichment analyses for each cluster were also referred to while identifying cell types.

### Protein–protein interaction network construction and identification of hub genes

The Search Tool for the Retrieval of Interacting Genes (STRING; http://string-db.org) (version 11.0) is an online analyzing tool that is used to draw functional interactions among multiple genes or proteins. This study used STRING to build a PPI network of the upregulated DEGs for each cell type. The results obtained in STRING were then loaded into Cytoscape (version 3.7.1), which is an open source software for visualizing PPI and combining PPI with attribute data. The hub genes for each cell type were screened based on the frequency of occurrence in the 12 algorithms from Cytoscape. No fewer than six times were deemed significant.

### Pseudotime analysis

Pseudotime analysis was conducted with Monocle in the R package (http://cole-trapnell-lab.github.io/monocle-release/, version 2). The annotated Seurat object was then converted into a Monocle object. Cells were arranged along a developmental axis to present their speculated trajectory location by using the orderCells function. The assumed developmental line was colored by cell types.

### Cell–cell interaction

Cell–cell interaction networks were drawn using the CellChat R package (https://htmlpreview.github.io/?https://github.com/sqjin/CellChat/blob/master/tutorial/CellChat-vignette.html). The annotated Seurat object was converted into a CellChat object. Ligands or receptors that were upregulated in one cell type were identified using the identify Over ExpressedGenes function, and gene expression data were then projected into the protein interaction network using the projectData function. The ligand–receptor interaction was screened as long as either the ligand or the receptor was overexpressed using the identify OverExpressedInteractions function. CellChat rendered each interaction a probability value and calculated the significance of the interaction. The calculation of the probability value not only took the expression matrix into consideration but also integrated the prior knowledge of the interaction.

### Single-cell regulatory network inference and clustering

SCENIC is a kind of algorithm that can be used to analyze the regulatory network with respect to transcriptional factors (TFs) and identify regulons in cells. The annotated Seurat object was imported to conduct SCENIC (version 0.9.1). The genes were filtered with default parameters, and a *Homo sapiens* specific database (hg19) was applied to use RcisTarget and SCENIC. The GENIE3 function was used to calculate the correlation between TFs and each regulon. Then, “runSCENIC” was used to speculate and validate the co-expression module. Finally, the activity of regulons was scored by the AUCell (area under the curve) algorithm that used a default threshold to binarize the specific regulons (“0” refers to “off” of TFs, while “1” indicates “on”).

## Results and discussion

### Cell-type annotation

**FIGURE 2:**
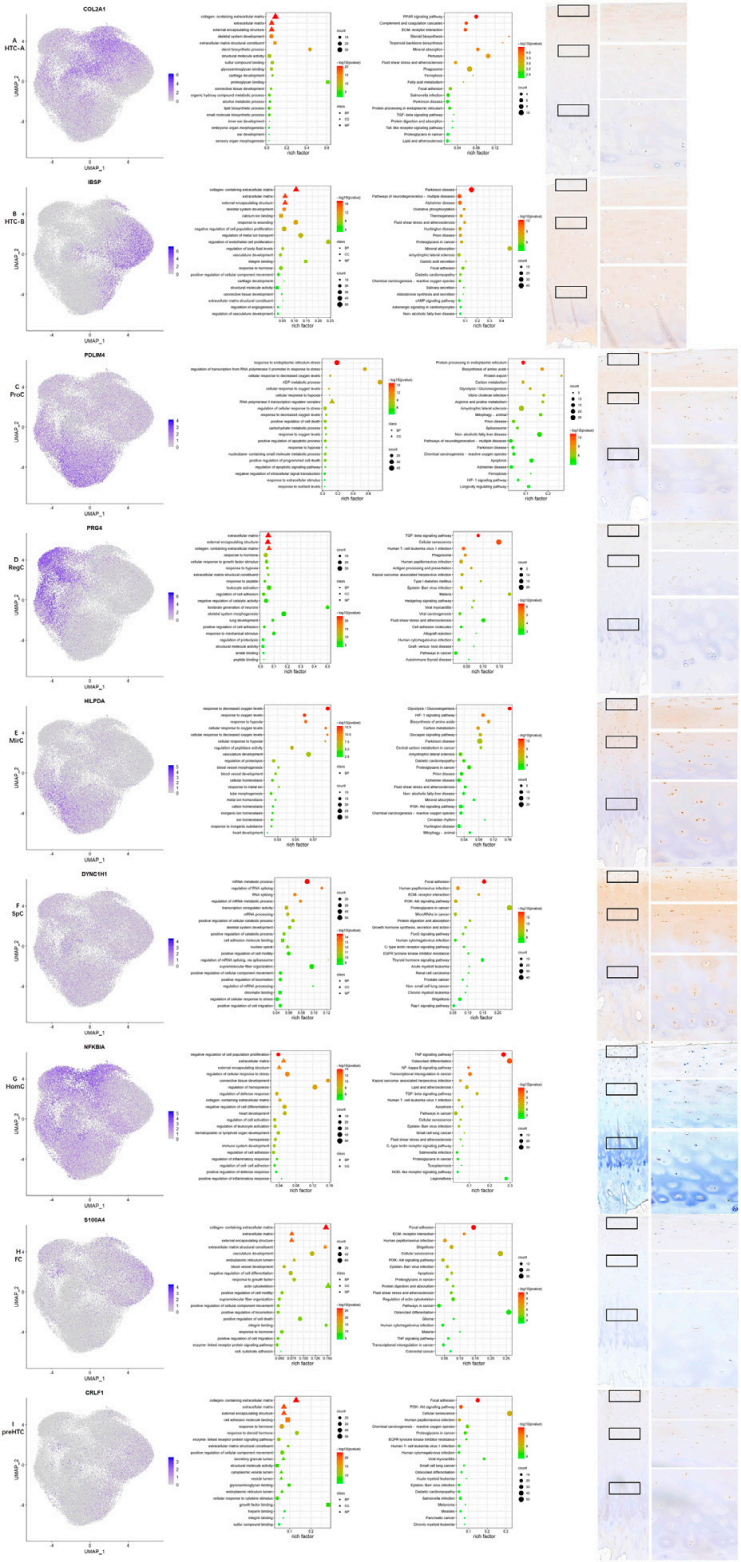
Results for specific markers, enrichment analysis and IHC for each cell type. **(A–I)** From left to right, the scattered plot for specific markers, GO enrichment analysis, KEGG pathway enrichment analysis, and IHC for specific makers.

In total, 65,835 cells were filtered and divided into 16 clusters (clusters 0–15). Basic information of quality control is shown in Figure 1B, and results for clustering and cell-type annotation are shown in Figures 1C,E; the distribution of each sample is shown in Figures 1D,G, the percentage of each cluster in the whole tissue is presented in Figure 1F, and the top 10 DEGs in each cluster are shown on the heatmap (Figure 1H). Results for pseudotime analysis are shown in Figures 1I,J. The scattered plot for identifiable markers and their distribution in IHC of each cell type indicated that all but HTC-B were dispersed in the superficial and middle regions of cartilage, while HTC-B was located in each part. GO and KEGG enrichment analyses for each cell type are shown in Figure 2, and detailed markers and enrichment analysis for each cell type are provided in Supplementary Tables S1–S3.

One previous study screened a chondrocyte subset and found that MT gene expression levels were high in cartilage samples obtained from Kashin–Beck patients (Wang et al., 2021), and the authors annotated it as HomCs. However, it is well known that MT genes encode proteins that are able to bind to metal ions, and GO enrichment analysis also suggested different functions compared with those of HomCs, as reported by Fuchou Tang et al. Therefore, the present study annotated them as a new chondrocyte subset—MirCs. It is reported that MT genes play an important role in protection from oxidative stress (Yao et al., 2015), and thus, MirCs may function in preventing mechanical loading-induced damage. Therefore, MirCs may be useful in managing force bearing on the talus. KEGG pathway enrichment analysis indicated that the HIF-1 signaling pathway was enriched in MirCs. It has been shown that metal ions have the potential to induce iron oxidation in hydroxylases and then stabilize HIF-1 (Kaczmarek et al., 2009). In addition, HIF-1 is a hypoxia-inducible factor that plays a key role in the response to hypoxia, which corresponds to the GO enrichment terms in MirCs. It is reported that HIF-1, as well as low oxygen levels, is an important stimulus in chondrocyte development (Huang and Hales, 2012), and therefore, MirCs may also be essential in these processes. SpCs were also a kind of new cell type found in this study. There were fewer pct1 of nearly all DEGs than pct2. In GSE157007 (https://www.ncbi.nlm.nih.gov/geo/query/acc.cgi?acc=GSE157007), a recent study published in Nature Aging identified a kind of aging cells from cord blood, and the number of pct1 of these aging cells was also smaller than that of pct2. SpCs may also be a kind of aging cells. Trajectory analysis also supported this speculation. GO terms for SpCs were listed according to the *p*-value instead of the number of top 20 markers, as shown in other cell types, since the specificity of SpCs markers was not significant. Regulating RNA splicing and cell movement processes was an important function for this cell type, which suggested the key role of the two functions in chondrocyte aging.

Similar to MirCs, GO terms and KEGG enrichment analysis indicated that ProCs also participated in response to the oxygen level and HIF-1. In addition, the major functions for ProCs also highlighted the regulation of cell death, which were different from those reported by Fuchou Tang et al.; these might suggest new functions for ProCs in the talus. The GO enrichment terms for HTC-A in this study were similar to those in the study conducted by Fuchou Tang et al., but GO terms such as ossification and mineralization, as elucidated by Fuchou Tang et al., were not found in HTC-B. GO terms for preHTCs were also quite different from those of the study by Fuchou Tang et al. Noticeably, it highlighted functions in response to the steroid hormone and heparin binding, which might serve as therapeutical targets for ankle cartilage diseases. GO terms for RegCs included responses to mechanical stress and hypoxia, as well as cellular growth and tissue development, suggesting that RegCs might assist MirCs and ProCs in managing external stress. HomCs, the most stable cell type for chondrocytes, should be present along the development line. However, trajectory analysis of this study showed that the number of HomCs started to increase with time. In addition, GO enrichment analysis also highlighted its function in immune regulation, which was quite different from results reported by Fuchou Tang et al., possibly suggesting a new microenvironment in the healthy talus. GO enrichment terms for FCs indicated that their major functions might be regulating the cell movement and actin processes, which were similar to those of the study conducted by Fuchou Tang et al. In addition, it is reported that the chondrocyte in which *PRG4* is highly expressed possesses characteristic for the progenitor, which may be potential functions for HomCs and RegCs in the talus (Saito, 2022). ECs and CPCs were not identified in this study, and expression levels of representative markers for the two subsets were low, indicating that the two chondrocyte cell types may be absent in the normal talus cartilage. Thus, chondrocyte subset identification in the talus may present a different microenvironment in coping with mechanical loading, which has not been explored.

### Cell–cell interaction

**FIGURE 3:**
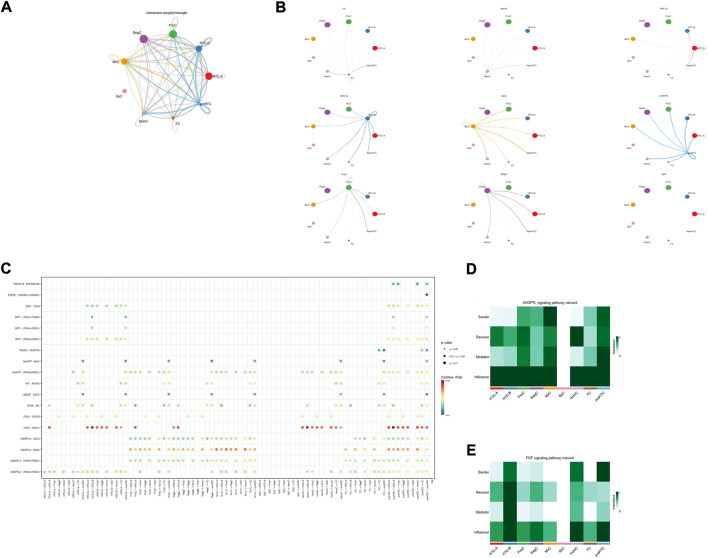
Results for the cell–cell interaction among cell types. **(A,B)** Interaction weight for cell–cell communication among each cell type. The size of the circle represents the ratio of each cell type; the width of the line represents the strength of cell–cell communication. **(C)** Interaction probability for each pathway among cell types; the size and color of the circle represent the *p*-value and probability, respectively. **(D,E)** Heatmap for the role of each cell type in different pathways; the color of the rectangle represents the extent of importance in each role.

Figures 3A,B showed the cell–cell interaction strength for each cell type. The bubble diagram (Figure 3C) and heatmap (Figures 3D,E) presented the significance of each signaling pathway and the role of each cell type. It has been demonstrated that *ANGPTL4* participates in matrix remodeling and is upregulated by responding to hypoxia in chondrocytes (Murata et al., 2009; Mathieu et al., 2014). However, *SDC2* is found to be involved in matrix homeostasis and cell adhesion (Wang et al., 2011). The strong interaction in the *ANGPTL4*-*SDC2* pathway, especially the important role of HTC-B, suggested that HTC-B might participate in matrix remodeling in the talus, which could be supported by its GO enrichment terms. The *FGF2*-*FGFR1* pathway is found to promote tissue repair (Figueroa et al., 2019). ProCs, MirCs, and RegCs were all involved in this pathway, which corresponded to the previous discussion that there existed similar GO terms among the three cell types. Considering that *FGF2* is also a hub gene for preHTCs, the four cell types may be involved in cartilage repair in healthy talus cartilage to combat mechanical force. Lastly, as shown in the bubble diagram, SpCs seemed to be inactive or independent compared with other cell types, which might explain the aging condition in SpCs. Briefly, in talus cartilage, HTC-B may participate in ECM remodeling, whereas ProCs, MirCs, RegCs, and preHTCs may be involved in tissue repair against force bearing. Future studies are needed to confirm these results.

### Single-cell regulatory network inference and clustering

**FIGURE 4:**
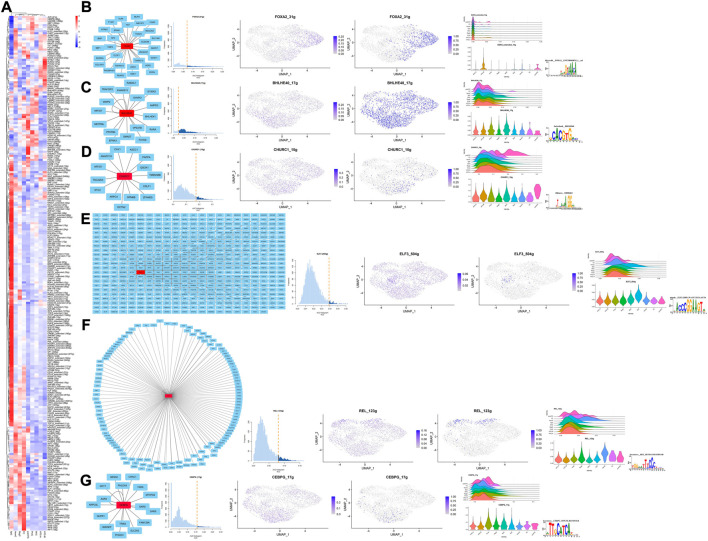
Results for SCENIC. **(A)** Heatmap presents the TFs’ activity in each cell type, respectively; the color of the rectangle indicates the relative expression levels for each TFs. **(B–G)** From left to right, the PPI network, AUC histogram, ridge plots, and violin plots, the potential targets of specific TFs in each cell type.

Results for SCENIC are summarized in Figure 4, and specific TFs for each cell type were screened according to the heatmap, scattered diagram, ridge plot, and violin plot. Their potential target genes were shown as PPI networks *via* Cytoscape, and the corresponding motifs are also shown in Figures 4B–G. It is well known that REL has a close interaction with NF-kB, which has the potential to confront stress (Hayden and Ghosh, 2004), suggesting potential functions of HomCs in managing mechanical loading. One recent study has found that *FOXA2* is an important TF for chondrocytes differentiating into hypertrophy (Bell et al., 2022), which corresponded to HTC-B. *BHELHE40* is reported to be a target of HIF-1 and also participates in chondrocyte development (Shen et al., 1997; Miyazaki et al., 2002; Shen et al., 2002), which is in accordance with the possible function of MirCs. *ELF3* is demonstrated to be a harmful gene in preventing OA progression and also participates in dysregulation of *COL2A1*, which corresponds to the potential role of SpCs (Otero et al., 2017; Miao et al., 2021). The roles of *CEBPG* and *CHURC1* in chondrocytes required further exploration. Briefly, specific TFs are mostly in line with the supposed function of their cell types and may be associated with the mechanical loading in talus cartilage.

### Identification of hub genes for each cell type

PPI networks for each cell type are provided in Supplemental Figures S1–S9. Hub genes for each cell type were identified according to 12 algorithms in Cytoscape and are listed in Table 2.

**TABLE 2:** Summarized hub genes for each cell type.

FC	HomC	HTC-A	HTC-B	SpC	preHTC	RegC	ProC	MirC
*FN1*	*NFKB1*	*COL2A1*	*NDUFS6*	*EP300*	*PIK3R1*	*GAPDH*	*GAPDH*	*GAPDH*
*GAPDH*	*NFKB2*	*DCN*	*ANXA2*	*THBS1*	*ANXA2*	*NFKBIA*	*JUN*	*ENO1*
*CCND1*	*BIRC3*	*LDLR*	*CAV1*	*HNRNPU*	*FN1*	*B2M*	*ATF4*	*FN1*
*CD44*	*FGF2*	*LUM*	*NDUFB7*	*KMT2A*	*CAV1*	*TIMP1*	*ENO1*	*GNB2L1*
*PSMB8*	*HIF1A*		*ACTB*	*MDM2*	*PTPN11*	*BMP2*	*DDIT3*	*LDHA*
*CAV1*	*S100B*		*BSG*	*VEGFA*	*DCN*	*ICAM1*	*XBP1*	*PKM*
*NFKBIA*	*SQSTM1*			*FOXO3*	*FGF2*	*NFE2L2*		*VEGFA*
				*KMT2C*	*PTRF*	*THBS1*		*PGK1*
				*POLR2A*		*VCAM1*		*FBL*

Because the number of hub genes for the nine cell types was relatively large, this study only screened hub genes that showed up more than once and speculated on their functions in the talus cartilage. *GAPDH* was identified as a hub gene for RegCs, ProCs, and MirCs, which suggested that response to hypoxia and the associated response to mechanical loading may be key functions for the three cell types, as well as the whole talus cartilage. In addition, GAPDH was also identified as a hub gene for FCs, possibly due to the involvement of cellular movement in GO terms. CAV1 was identified as a hub gene for HTC-B, preHTCs, and FCs. It is reported that *CAV1* is a kind of senescent marker for chondrocytes (Toh et al., 2016), which indicates the possible condition for the three chondrocyte subsets. DCN is reported to enhance the function of the aggrecan and resist micromechanics in the ECM (Maly and Andres Sastre, 2021), which, as a hub gene for HTC-A and preHTCs, may suggest a possible function of the two cell types in managing mechanical loading. As discussed previously, *FGF2* was associated with tissue repair, and HomCs and preHTCs were two cell types mainly located at late stages, which might function as repairing cartilage in the talus. Scientists have verified that FN1 participates in cell adhesion, and mechanical stimulation can reduce its expression (Steinecker-Frohnwieser et al., 2014; Zhang et al., 2021). However, in FCs, preHTCs and MirCs, it was identified as a hub gene; possibly the three cells manage to cope with hypoxia and mechanical loading, especially MirCs. *ANXA2* served as ECM mineralization, and its role as a hub gene for HTC-B indicated the functions of HTC-B elucidated by Fuchou Tang et al.. *ENO1* was a hub gene for ProCs and MirCs, and *NFKBIA* was a hub gene for ProCs and preHTCs. One study has elucidated that *ENO1* and *NFKBIA* are important factors in OA progression (Bhosale and Richardson, 2008), which may indicate a double-sided impact of ProCs, MirCs, and RegCs. *VEGFA*, a hub gene for MirCs and SpCs, and *THBS1*, a hub gene for SpCs and RegCs, have been shown to be involved in angiogenesis (Zhan and Cai, 2019; Zhao et al., 2020), which can be induced by mechanical loading (Beckmann et al., 2014). Briefly, the 10 hub genes may indicate special functions for identified cell types and the whole talus cartilage, especially in managing mechanical loading.

## Limitations and conclusions

To the best of our knowledge, as the first study that used scRNA-seq to explore chondrocyte atlas in the talus, this study has several limitations. First, the annotation database for chondrocyte subsets was absent. This article mainly referred to a study conducted by Fuchou Tang concerning OA chondrocytes, which may have influenced the results of this study. Second, quite a number of the contents in the Discussion section were speculated upon, and a validation study is lacking. Third, although the markers selected in IHC were the most specific in the scattered diagram, several of the markers were still representative of their cell type. Lastly, due to the memory constraints, 15,000 cells and 4,500 cells are chosen randomly to conduct pseudotime analysis and SCENIC, respectively, which may have an impact on the results, although the randomized method has been widely accepted.

In conclusion, this study applied scRNA-seq to demonstrate atlas for chondrocyte subsets from the healthy talus cartilage, which indicated that MirCs, ProCs, and RegCs might work together to manage the effect of mechanical loading on the talus cartilage, and cell–cell interactions and transcription factors were also involved in this background. The identified and speculated novel microenvironment may pose different directions in chondrocyte composition, development, and metabolism in the talus, which may serve as therapeutical targets for ankle cartilage diseases. Future researchers should try to verify the cellular and molecular mechanisms found in this study.

## Data Availability

The data presented in the study are deposited in the GEO repository, accession number GSE216578. Any further inquiries can be directed to the corresponding author.

## Supplementary material

SUPPLEMENTAL FIGURE S1–S9 PPI networks for DEGs in each cell type. Figure 1, FC, Figure 2, HomC, Figure 3, HTC-A, Figure 4, HTC-B, Figure 5, MirC, Figure 6, preHTC, Figure 7, ProC, Figure 8, RegC, Figure 9, SpC.

SUPPLEMENTAL TABLE S1 Detailed DEGs for each cell type.

SUPPLEMENTARY TABLE S2 Detailed results for GO enrichment analysis for each cell type.

SUPPLEMENTARY TABLE S3 Detailed results for KEGG pathway enrichment analysis for each cell type.
